# Physician review of image registration and normal structure delineation

**DOI:** 10.1002/acm2.13031

**Published:** 2020-09-28

**Authors:** William Tyler Turchan, Ritu Arya, Robert Hight, Hania Al‐Hallaq, Michael Dominello, Dan Joyce, Bradley P. McCabe, Anne R. McCall, Eugenia Perevalova, Christopher Stepaniak, Kamil Yenice, Jay Burmeister, Daniel W. Golden

**Affiliations:** ^1^ Department of Radiation and Cellular Oncology The University of Chicago Chicago IL USA; ^2^ Department of Oncology Division of Radiation Oncology Wayne State University Karmanos Cancer Institute Detroit MI USA

**Keywords:** image registration, organs at risk, patient safety, quality assurance, radiotherapy

## Abstract

**Introduction:**

Image registration and delineation of organs at risk (OARs) are key components of three‐dimensional conformal (3DCRT) and intensity‐modulated radiotherapy (IMRT) treatment planning. This study hypothesized that image registration and OAR delineation are often performed by medical physicists and/or dosimetrists and are not routinely reviewed by treating physicians.

**Methods:**

An anonymous, internet‐based survey of medical physicists and dosimetrists was distributed via the MEDPHYS and MEDDOS listserv groups. Participants were asked to characterize standard practices for completion and review of OAR contouring, target volume contouring, and image registration at their institution along with their personal training in these areas and level of comfort performing these tasks. Likert‐type scales are reported as Median [Interquartile range] with scores ranging from 1 = “Extremely/All of the time” to 5 = “Not at all/Never.”

**Results:**

Two hundred and ninety‐seven individuals responded to the survey. Overall, respondents indicated significantly less frequent physician review (3 [2–4] vs 2 [1–3]), and less confidence in the thoroughness of physician review (3 [2–4] vs 2 [1–3], *P* < 0.01) of OAR contours compared to image registration. Only 19% (95% CI 14–24%) of respondents reported a formal process by which OAR volumes are reviewed by physicians in their clinic. The presence of a formal review process was also associated with significantly higher perceived thoroughness of review of OAR volumes compared to clinics with no formal review process (2 [2–3] vs 3 [2–4], *P* < 0.01).

**Conclusion:**

Despite the critical role of OAR delineation and image registration in the 3DCRT and IMRT treatment planning process, physician review of these tasks is not always optimal. Radiotherapy clinics should consider implementation of formal processes to promote adequate physician review of OARs and image registrations to ensure the quality and safety of radiotherapy treatment plans.

## 1 INTRODUCTION

The advent of three‐dimensional conformal radiotherapy (3DCRT) and intensity‐modulated radiotherapy (IMRT) now allows delivery of radiotherapy in a highly conformal manner. A key component of the 3DCRT and IMRT treatment planning process is the accurate delineation of target volumes (TVs) and surrounding organs at risk (OARs). While the appropriate demarcation of TVs is critical to ensuring that patients’ disease is adequately treated, accurate OAR delineation is crucial for inverse treatment planning and ensures that the dose to OARs is correctly determined.

Despite the critical role of TV and OAR accuracy in delivering safe and effective treatment with 3DCRT and IMRT, delineation of these structures is often associated with a steep learning curve with significant potential for error. For example, upon central review of the contours from RTOG 0529 it was found that the small and large bowel were incorrectly contoured 60% and 45% of the time, respectively.^1^ In an attempt to standardize the way in which TVs and OARs are contoured, numerous consensus guidelines and contouring atlases have been developed.^2^, ^3^, ^4^, ^5^, ^6^, ^7^ Another strategy to help ensure TVs and OARs are accurately delineated is image registration, during which diagnostic image sets are fused to the image sets obtained at the time of simulation. Although image registration can improve the ability to accurately demarcate volumes of interest, this is dependent upon the accuracy of the registration of diagnostic image sets to the image sets obtained at the time of simulation, a process that can have many sources of error.^8^

Initially published by ASTRO in 2012, *Safety is No Accident*, provides a framework to help ensure the quality and safety of radiotherapy treatments. The most recent version of *Safety is No Accident* specifically defines the roles and responsibilities of members of the radiotherapy team and states that among other tasks, the treating radiation oncologist has the responsibility to “define the [TVs] on the images obtained during simulation,” “confirm registration,” and “review [OARs] delineated by planning staff for accuracy.” Furthermore, it is recommended that “after the radiation oncologist defines [TVs] and normal tissues, when possible, another physician should review and confirm the contours before treatment planning begins.”^9^

This study hypothesized that in line with the framework provided by *Safety is No Accident*, TVs are delineated by the treating physician in the majority of clinics; while image registration and OAR contouring are typically performed by a dosimetrist or medical physicist. However, we also hypothesized that the accuracy of OAR contours and image registrations are not routinely reviewed by treating physicians and that formal peer review of these steps prior to the treatment planning process is suboptimal.

## 2 MATERIALS AND METHODS

### 2.A Survey

An anonymous, internet‐based survey was developed with input from radiation oncologists, medical physicists, and dosimetrists at multiple institutions. Questions in the survey included multiple choice, Yes‐No, multiple‐item Likert‐type scale (1 = Extremely/All of the time, 2 = Quite/Most of the time, 3 = Moderately/Some of the time, 4 = Slightly/Rarely, and 5 = Not at all/Never), and free‐response formats.

#### 2.A.1 Demographics/practice patterns

The survey (File S1) was divided into four main sections. Branching logic was used in multiple instances to ask relevant follow‐up questions and to allow participants to elaborate on specific responses to questions. The first section of the survey consisted of general questions to establish the participant’s job title, clinical role, and clinic demographics.

#### 2.A.2 OARs

The second section consisted of two subsections focused on OAR delineation. In the first subsection, participants were asked to list the job title of individuals who routinely enter OAR volumes in their clinic. Participants who enter OAR volumes as part of their clinical responsibilities were subsequently asked to characterize their confidence in their ability to accurately contour OARs in general as well as for specific commonly contoured OARs. The list of specific OARs that participants were asked to rate their confidence contouring was developed from several institutional scorecards used for plan evaluation with subsequent input from physicians, dosimetrists, and medical physicists who contributed to the survey design. Participants were also asked about any formal or informal training they had received for contouring OARs. In the second subsection, participants were asked to characterize the review of OARs by the physicians at their clinic including the frequency and thoroughness of physician review of OAR volumes along with the presence or absence of a formal process for physician review of OAR volumes.

#### 2.A.3 Image registration

The third section focused on image registration; in the first subsection participants were asked to detail the individuals at their clinic who routinely register diagnostic image sets with image sets obtained at the time of simulation and if applicable were subsequently asked to characterize their confidence in their ability to register images accurately. The second subsection focused on physician review of image registration.

#### 2.A.4 TVs

Participants were also asked to describe the individuals responsible for entry of TVs at their institution; however, given the scope of this study, participants were not asked to characterize TV entry or evaluation further. The final section of the survey asked participants to characterize how frequently changes or errors in components of the 3DCRT and IMRT treatment planning process necessitating changes to the treatment plan are discovered in their clinic.

### 2.B Survey distribution

The survey was distributed via an e‐mail message sent to the MEDPHYS and MEDDOS listserv groups (File S2) containing a link to the survey. These listserv groups are intended to facilitate communication among medical physicists and dosimetrists, respectively. At the time of this survey, the MEDPHYS and MEDDOS listserv groups had approximately 6200 and 1500 subscribers, respectively.^10^ Survey invitations were initially sent on February 4, 2019. The survey was estimated to take 10–15 min to complete. Respondents were permitted to save their responses and return later to complete the survey. Study data were collected and managed using Research Electronic Data Capture (REDCap). REDCap is a secure, web‐based application designed to support data capture for research studies, providing: (a) an intuitive interface for validated data entry; (b) audit trails for tracking data manipulation and export procedures; (c) automated export procedures for seamless data downloads to common statistical packages; and (d) procedures for importing data from external sources.^11^ These electronic data capture tools are hosted at the University of Chicago. Following the initial e‐mail message, a reminder message was sent to the listservs on February 21, 2019. The survey was closed to responses on February 28, 2019.

### 2.C Statistics

Statistical analysis was performed using JMP®, Version 14 (SAS Institute Inc., Cary, NC). All tests were two‐sided. The χ^2^ test was used to compare among discrete variables and the t test among continuous variables. Differences between medians were assessed using the Wilcoxon rank sum or Kruskal–Wallis tests. Likert‐type results are reported as median [interquartile range]. Percentages are reported as percent [95% confidence interval]. The University of Chicago Institutional Review Board determined this project to be exempt from review.

## 3 RESULTS

### 3.A Demographics/practice patterns

Two hundred and ninety‐seven individuals responded to the survey (estimated response rate 4%). Of those who responded, 213 (74%) were medical physicists, 69 (24%) were dosimetrists, and 4 (1%) were medical physics residents or postdoctoral fellows. Further details regarding the demographics of the respondents are given in Table 1.

**Table 1:** Demographics of survey respondents.

Job title	n (%)
Medical physicist	213 (74%)
Dosimetrist	69 (24%)
Medical physics resident	4 (1%)
Continent	
Asia	28 (10%)
Africa	7 (2%)
Europe	41 (14%)
North America	199 (70%)
Oceania	4 (1%)
South America	6 (2%)
Clinic type	
Academic medical center main campus	58 (20%)
Free‐standing radiation oncology clinic	51 (18%)
Government‐affiliated hospital	19 (7%)
Hospital‐based community practice	140 (49%)
Network site for academic medical center	16 (6%)

Note

Percentages may not add up to 100% in all instances as a result of rounding.

When asked to characterize the process of image registration and OAR and target delineation, respondents reported that dosimetrists and/or medical physicists are solely responsible for importing the primary image set, registering diagnostic images with the primary image set, and entering OAR contours at their clinics 95% (95% CI 93–97%), 89% (95% CI 86–92%), and 63% (95% CI 58–68%) of the time, respectively. 69% (95% CI 64–74%) responded that, at their clinic, only physicians enter TVs. Further details regarding which member(s) of the radiotherapy team perform these tasks in the represented clinics are shown in Table 2.

**Table 2:** Frequency with which tasks are performed by job title.

Image importing	n (%)
Treating/attending physicians	10 (3%)
Resident physicians	7 (2%)
Dosimetrists	207 (70%)
Medical Physicists	86 (29%)
Radiation Therapists	42 (14%)
Other	0 (0%)
Image registration	
Treating/attending physicians	30 (10%)
Resident physicians	12 (4%)
Dosimetrists	159 (54%)
Medical Physicists	143 (48%)
Radiation Therapists	9 (3%)
Other	1 (0.3%)
OAR contouring	
Treating/attending physicians	92 (31%)
Resident physicians	48 (16%)
Dosimetrists	214 (72%)
Medical Physicists	78 (26%)
Radiation Therapists	23 (8%)
Other	4 (1%)
Target volume entry	
Treating/attending physicians	197 (66%)
Resident physicians	47 (16%)
Dosimetrists	23 (8%)
Medical Physicists	14 (5%)
Radiation Therapists	4 (1%)
Other	1 (0.3%)

OAR, Organ at risk.

### 3.B Image registration

One hundred and eighty‐four (81%, 95% CI 77–85%) respondents reported performing registration of diagnostic images with the primary image set as part of their clinical responsibilities. Overall, respondents are confident in their ability to appropriately register diagnostic images with the primary image set (Likert‐type score 2 [1–2]). Median confidence of respondents in their ability to appropriately register diagnostic images with the primary image set varied significantly on Likert‐type scale by diagnostic imaging modality with higher confidence for CT than for MRI or PET: 1 [1–2], 2 [1–2], and 2 [1–2] (*P* < 0.01). Of the respondents who report performing registration of diagnostic images with the primary image set as part of their clinical responsibilities, 128 (67%, 95% CI 60‐–4%) indicated that they relied on the assistance of automated image registration software for “all” or “most” of the image registration that they perform (Likert‐type score 2 [2–3]), and confidence on Likert‐type scale in the accuracy of registration performed with automated software was rated as a 2 [2–3].

When asked to characterize how often physicians at their institution reviewed diagnostic image registration performed by others, the median Likert‐type score (from 1 = always to 5 = never) was 2 [1–3]. When asked to rate their confidence in the thoroughness of physician review of diagnostic image registration, respondents gave a median Likert‐type score of 2 [1–3]. While most respondents indicated that in cases where they are uncertain of the accuracy of image registration they explicitly ask the treating physician to review the registration, 19 (9%, 95% CI 5–13%) indicated that they do not ask the treating physician to review the registration. When asked to characterize the reason that they do not ask the treating physician to review image registration when they were uncertain of the accuracy, respondents most commonly indicated that they did not feel as though the treating physician would review the registration thoroughly and/or asking the physician to do so would not improve the accuracy.

### 3.C Contouring

Twenty‐two (10%, 95% CI 6–15%) and 152 (56%, 95% CI 60–62%) respondents indicated that they contour TVs and OARs, respectively, as part of their clinical responsibilities. Overall, the respondents are confident in their ability to contour OARs (2 [1.75–2]). Respondents were given a list of 33 frequently contoured OARs and asked to indicate whether they contoured each OAR as part of their clinical responsibilities and, if so, to rate their confidence in their ability to do so accurately (Table 3 **)**. Respondents indicated they had the most difficulty contouring the brachial plexus (3 [3–5]).

**Table 3:** Frequency and confidence contouring common organs at risk. (Likert‐type scale 1 = “Extremely” to 5 = “Not at all”).

OAR	Respondents reporting contouring this OAR n (%)	Confidence contouring OAR on Likert‐Type Scale (Median [IQR])
Bladder	127 (97%)	1 [1‐1]
Eye	130 (99%)	1 [1‐1]
Femoral Head	126 (96%)	1 [1‐1]
Kidney	126 (96%)	1 [1‐1]
Lens	127 (97%)	1 [1‐1]
Lung	129 (98%)	1 [1‐1]
Mandible	124 (95%)	1 [1‐1]
Ribs	119 (91%)	1 [1‐1]
Spinal Cord	130 (99%)	1 [1‐1]
Chest Wall	120 (92%)	1 [1‐2]
Esophagus	126 (96%)	1 [1‐2]
Heart	126 (96%)	1 [1‐2]
Liver	124 (95%)	1 [1‐2]
Optic Nerve	123 (94%)	1 [1‐2]
Prostate	91 (69%)	1 [1‐2]
Rectum	123 (94%)	1 [1‐2]
Stomach	116 (88%)	1 [1‐2]
Brainstem	126 (96%)	2 [1‐2]
Great Vessels	104 (79%)	2 [1‐2]
Parotid Gland	114 (87%)	2 [1‐2]
Cauda Equina	106 (81%)	2 [1‐3]
Cochlea	111 (85%)	2 [1‐3]
Large Bowel	112 (86%)	2 [1‐3]
Optic Chiasm	119 (91%)	2 [1‐3]
Proximal Bronchial Tree	104 (79%)	2 [1‐3]
Penile Bulb	101 (77%)	2 [1‐3]
Small Bowel	113 (86%)	2 [1‐3]
Submandibular Gland	104 (79%)	2 [1‐3]
Uterus	80 (61%)	2 [1‐3.25]
Duodenum	99 (76%)	3 [2‐3.25]
Sacral Plexus	68 (52%)	3 [2‐4]
Ovary	72 (55%)	3 [3‐4]
Brachial Plexus	92 (70%)	3 [3‐5]

IQR, interquartile range; OAR, organ at risk.

Ninety‐five individuals (73%, 95% CI 66–80%) who contour OARs as part of their clinical duties indicated that they had previously had some type of either formal or informal training contouring normal structures. Respondents who indicated that they had previously had some type of training contouring OARs were asked to characterize the type(s) of training that they had received and asked to indicate how helpful they perceived each type of training to be (Table 4). Respondents who had previous training contouring OARs most frequently indicated receiving some type of informal training as part of either their current and/or former job, as indicated by 65 (71%, 95% CI 62–80%) respondents. Individuals who had previous training contouring OARs were significantly more confident in their ability to accurately contour OARs compared to individuals who had not had training previously (2 [1–2] vs 2 [2–3]; *P* < 0.01). A trend toward increasing confidence in ability to accurately contour OARs with increased frequency of OAR contouring was also noted, among respondents who contour “all” (2 [1–2]), “most” (2 [1–2]), “some” (2 [2–2]), and “a few” (2 [2–3.25]) of the normal structures in the treatment plans they work on (*P* = 0.06).

**Table 4:** Frequency and effectiveness of training previously received for contouring organs at risk. (Likert‐type scale 1 = “Extremely” to 5 = “Not at all”).

Training type	n (%)	Effectiveness of training on Likert‐Type Scale (Median [IQR])
Formal training as part of the curriculum during classroom education	26 (27%)	2 [1–3]
Formal training as part of the curriculum during postgraduate training/medical physics residency	17 (18%)	2 [1.5–3]
Formal training as part of current job	14 (15%)	2 [1–2.25]
Formal training as part of a former job	13 (14%)	2 [1–2]
Attending a talk on contouring normal structures at a professional society meeting	38 (40%)	2 [2–3]
Informal "on the job" training as part of current job	49 (52%)	2 [1.5–3]
Informal "on the job" training as part of a former job	39 (41%)	2 [1–2]
Other	9 (9%)	1 [1–2]

IQR, interquartile range.

Of the respondents who reported contouring OARs as part of their clinical responsibilities, 87 (67%, 95% CI 60–74%) indicated that they “never” or “rarely” use auto‐segmentation or other automated processes to assist in contouring OARs (Likert‐type score 4 [3–4]). When asked to indicate their confidence in the ability of auto‐segmentation and other automated processes to assist in the accurate delineation of OARs, respondents gave a median Likert‐type score of 3 [3–4]. On the whole, respondents indicated significantly less frequent utilization (*P* < 0.01) and lower confidence in the accuracy (*P* < 0.01) of automated methods to assist in OAR delineation compared to image registration.

When asked to characterize how often physicians at their institution review OAR contours on a Likert‐type scale responses were mixed (3 [2–4]). Median confidence in the thoroughness of OAR volume review performed by physicians was 3 [2–4]. Overall, respondents indicated significantly less frequent physician review (*P* < 0.01) and less confidence in the thoroughness of the review performed (*P* < 0.01) of OAR contours compared to image registration. Confidence in the thoroughness of the review of OAR contours and image registration did not differ significantly with respect to job title, clinic type, or geographic location (data not shown).

Only 37 (19%, 95% CI 14–24%) individuals indicated a formal process by which OAR volumes are reviewed by physicians at their clinic. Rates of formal review did not differ significantly by clinic type (*P* = 0.20) or geographic location (*P* = 0.10). Physician review of OAR contours occurred significantly more frequently in clinics where a formal review process was in place compared to clinics where no formal review process was in place (1 [1–2] vs 3 [2–3]; *P* < 0.01). Moreover, 32 (20%, 95% CI 15–25%) individuals at clinics without a formal review process stated that physicians “rarely” or “never” review OAR volumes, while no individuals (0%, 95% CI 0–3%) at clinics with a formal review process reported this. The presence of a formal review process was also associated with significantly higher perceived thoroughness of review of OAR volumes compared to clinics with no formal review process (2 [2–3] vs 3 [2–4]; *P* < 0.01). Moreover, errors in OAR delineation necessitating changes to the treatment plan were more likely to be caught prior to patients beginning treatment in clinics with a formal review process in place (n = 13, 35%, 95% CI 20–50%) compared to clinics with no formal review process in place (n = 26, 16%, 95% CI 10–22%; *P* = 0.02).

## 4 DISCUSSION

As hypothesized, this study found that, in line with the framework provided by *Safety is No Accident*, image registration and OAR delineation are performed primarily by dosimetrists and/or medical physicists in the majority of clinics surveyed. Accurate image registration is critical to the 3DCRT and IMRT treatment planning process to ensure accurate delineation of target and avoidance structures, while accurate OAR characterization ensures that the dose to each avoidance structure of interest can be correctly determined and evaluated by the treating physician. Interobserver variability in these aspects of the treatment planning process has been demonstrated previously and can be both dosimetrically and clinically significant.^5^, ^7^, ^11^ Given this, *Safety is No Accident* recommends that the treating radiation oncologist reviews image registration and OAR delineation performed by others to ensure the accuracy of these tasks.

As hypothesized, respondents indicated deficiencies in the frequency and thoroughness of physician review of image registration and OAR contours. Moreover, only 19% (95% CI 14–24%) of respondents reported a formal process by which OAR volumes are reviewed by physicians in their clinic, creating a potential quality and safety gap for patients receiving radiotherapy. This gap has been reported to affect patient care according to the Radiation Oncology Incident Learning System (RO‐ILS) database, which identified “problematic plan” errors including “poor image fusion” and errors related to the target and normal structures.^12^ Moreover, multiple studies performing failure mode and effect analysis (FMEA) have identified issues with inaccurate image registration and volume delineation as potential threats to the quality and safety of the care delivered in radiotherapy clinics.^13^, ^14^

When asked to characterize their confidence in their ability to accurately delineate specific normal structures from a list of common OARs, respondents’ confidence significantly varied based on the OAR, as shown in Table 3. While respondents were uniformly confident in their ability to accurately contour structures such as the lung and the bladder, they acknowledged uncertainty in their ability to accurately contour OARs such as the duodenum and the sacral plexus. Most notably, despite 70% (95% CI 62–78%) of respondents stating that they are responsible for contouring the brachial plexus when clinically applicable, 21% (95% CI 13–29%) acknowledged being “not at all comfortable” with their ability to do so accurately. This high rate of uncertainty combined with the possibly debilitating and irreversible consequences of brachial plexopathy underscores the potential for adverse events as a result of inaccurate delineation of OARs and the need for review of avoidance structures by the treating physician.^15^

Given the critical nature of accurate OAR delineation to ensure safety and quality in radiotherapy treatment plans, *Safety is No Accident* recommends that all physicians should review OARs delineated by dosimetrists and medical physicists to ensure the accuracy of these contours. This study indicates that physician review of OAR contours is suboptimal among the represented clinics and that, on average, respondents were only “moderately” confident in the thoroughness of physician review of OAR contours. One potential way to improve physician review of OAR contours is the implementation of a formal process for OAR review within clinics. Our results demonstrate that physician review of OAR contours, as judged by medical physicists and dosimetrists, is significantly more frequent and thorough in clinics with a formal review process in place and the presence of a formal review process significantly increases the number of treatment plan changes attributed to inaccurate OAR delineation that are caught prior to patients starting treatment. Despite this, only 19% (95% CI 14–24%) of respondents indicated the presence of such a process in their clinic, providing a potential area where the quality and safety of the radiotherapy treatment process could be improved.

Respondents who indicated the presence of a formal review process in their clinic were asked to further characterize this process. Most responses described a system in which the dosimetrist and/or medical physicist contours OARs and the OARs are then labeled in some manner to indicate that they have not yet been reviewed. The treating physician subsequently reviews the OARs and then changes the label to indicate that they have reviewed and verified the accuracy of the OAR contours. Moreover, a few respondents indicated the presence of prospective contouring rounds in their clinic, during which physician peer review of the clinical plan as well as OAR and TV contours is performed. This practice is in line with recommendations in *Safety is No Accident* and has been shown to improve peer review and treatment standardization and decrease delays in treatment as a result of modifications that need to be made to the treatment plan after the treatment planning process has been completed.^16^, ^17^

Aside from improving physician review, another potential strategy to improve OAR accuracy is the implementation of additional training for staff who contour OARs as part of their clinical responsibilities. Respondents who had previous training contouring OARs were significantly more confident in their ability to accurately delineate normal structures. Interestingly, among those indicating “other” types of training received (Table 4), several respondents indicated that they had received training via online contouring modules and these were most commonly indicated to be “extremely” (Likert‐type score of 1) effective. An example of one such program is The Anatomy and Radiology Contouring Bootcamp, which has been created with the goal of educating radiation oncology residents to accurately delineate TVs as well as normal structures;^18^ although, given the frequency with which medical physicists and dosimetrists were responsible for contouring OARs in this study, similar programs should be extended to these members of the radiotherapy team. Moreover, the current standards for accreditation in these fields should be re‐examined and potentially modified with an increased emphasis upon knowledge of normal anatomy and accurate contouring of OARs to reflect the significant degree of OAR delineation that is performed by these individuals.

Compared to OAR delineation, physician review of diagnostic image registration was rated as more regular and thorough among respondents. While this is comforting, it is troubling that 9% (95% CI 5–13%) of respondents indicated that in situations that they feel unsure about the accuracy of the image registration they do not ask the treating physician to review the registration and that the most common reason for this was lack of confidence in the thoroughness/effectiveness of the physician’s review. Given the demonstrated increase in thoroughness of physician review following implementation of formal review processes, incorporation of review of diagnostic image registration as part of prospective planning rounds should also be considered.

It should also be noted that while physicians were indicated to be responsible for contouring TVs in most cases, 31% (95% CI 26–36%) of respondents indicated that nonphysicians enter TVs in their clinics in some cases. However, given that characterization of the methods used to delineate TVs was not a focus of this study, this was not investigated further here and it is possible that some of these responses reflect nonphysician members of the radiotherapy team performing simple geometric expansions, such as expansion of the clinical target volume to make a planning target volume. Nonetheless, given that appropriate delineation of TVs requires understanding of anatomy and pathophysiology and the ability to synthesize this information with radiographic and clinical exam correlates, we recommend that, in line with recommendations made in *Safety is No Accident*, treating physicians should be solely responsible for delineation of TVs.

Several potential limitations should be considered when interpreting the results of this study. Most notably, the population of respondents in our study consisted of 297 individuals on the MEDPHYS and MEDDOS listserv groups. The MEDPHYS and MEDDOS listserv groups had over 6,200 and 1,500 subscribers, respectively, at the time of this survey.^10^ Under the assumption that the overlap in subscription to these two groups is minimal, this represents a response rate of approximately 4%. Although the potential for nonresponder bias increases when response rates fall below expected levels, it is important to note that expected response rate for a survey can vary significantly based on the group surveyed and the survey modality.^19^ Upon review of surveys distributed on these listservs between February 6, 2013 and January 2, 2018 Kisling et al reported a mean number of respondents of 63 and a maximum of 183 respondents,^10^ supporting the conclusion that the response rate for this survey was not uncharacteristic of the surveyed population. Regardless, the possibility exists that the cohort of respondents is biased with regard to their experiences and perceptions compared to the entire population of practicing medical physicists and dosimetrists and consequently our results overestimate the frequency of lapses in physician review of OAR contours and image registration. Moreover, it is important to note the possibility that the thoroughness of physician review of OAR contours and image registration, as perceived by medical physicists and dosimetrists, may not always correlate with how well physicians reviewed these aspects of the treatment planning process. Nonetheless, given the possibly disastrous results of inaccurate OAR delineation and/or image fusion, the individual cases of seemingly inadequate physician review of these processes identified in this report highlight a significant potential quality and safety gap.

## 5 CONCLUSION

Despite the critical role of OAR delineation and image registration in the 3DCRT and IMRT treatment planning process, physician review of these tasks, as reported by an international cohort of surveyed dosimetrists and medical physicists, is at times suboptimal. Radiotherapy clinics should consider the use of a formal review processes for OAR delineation and image registration to ensure the quality and safety of treatment plans, the implementation of which has been incorporated into a recently described framework for promoting safety and quality in radiotherapy clinics.^20^ Another potential strategy to improve the quality and safety of the treatment delivered by radiation therapy clinics is the implementation of standardized training programs for individuals who contour normal structures and perform image registration as part of their clinical responsibilities. Further studies to develop optimal training and systems‐based practices for OAR delineation and image registration are underway. Moreover, further studies are warranted to characterize physicians’ perception of this process and objectively evaluate the extent and quality of physician review of OARs and image registration along with how well these measures correlate with accuracy of OAR delineation and image registration.

## CONFLICTS OF INTEREST

Daniel W. Golden MD MHPE is a manager of RadOncQuestions.com and HemOncReview.com and has funding from the National Institutes of Health, Radiation Oncology Institute, and Bucksbaum Institute for Clinical Excellence. The authors declare no other relevant conflict of interest.

## Supporting information


**File S1.** Survey distributed to medical physicists and dosimetrists.

**File S2.** Invitation e‐mail sent to the MEDPHYS and MEDDOS listserv groups with the link to the survey.

**Caption**
