# Supplementary material for: Physician review of image registration and normal structure delineation
Source: J Appl Clin Med Phys. 2020 Sep 28;21(11):80–7. doi: 10.1002/acm2.13031 (PMC7701106; doi:10.1002/acm2.13031)
Supplement: Supplementary file 1 — File S1. Survey distributed to medical physicists and dosimetrists. [file ACM2-21-80-s001.pdf]

International Normal Structure, Target Volume, and Fusion Review Survey

Dear Colleague,

Thank you for taking time out of your busy schedule to participate in this survey. The goal of this survey is to characterize standard practices of normal structure contour delineation and reivew for 3D and IMRT treatment planning at the national level.

We estimate that this survey will take you approximately 10-15 minutes to complete, but feel free to spend longer.

Your responses are anonymous. We appreciate your thoughtful and honest answers.

Questions or comments can be addressed to XXX

Thank you in advance for taking the time to fill out this survey.

What is your job title?

◯ Medical Physicist
◯ Dosimetrist
◯ Other

What is your job title?

____

Which setting best describes your clinic?

◯ Academic medical center main campus
◯ Network site for academic medical center
◯ Hospital-based community practice
◯ Government-affiliated hospital
◯ Free-standing radiation oncology clinic
◯ Other

What setting best describes your clinic?

____

What is the geographic location of your clinic?

◯ Asia
◯ Africa
◯ Europe
◯ North America
◯ Oceania
◯ South America

What country is your clinic located in?

◯ United States of America
◯ Mexico
◯ Canada
◯ Other

What country is your clinic located in?

____

Normal Structure Contours

Who imports the primary image sets obtained from simulation scans into the treatment planning software at your clinic?
(Check all that apply.)

☐ Attending physicians
☐ Resident physicians
☐ Dosimetrists
☐ Medical physicists
☐ Radiation therapists
☐ Other

Who else imports primary image sets obtained from the simulation scan into the treatment planning software?

____

Who contours normal structures at your clinic?
(Check all that apply.)

☐ Attending physicians
☐ Resident physicians
☐ Dosimetrists
☐ Medical physicists
☐ Radiation therapists
☐ Other

Who else contours normal structures at your clinic?

____

Do you contour normal structures as part of your clinical responsibilities?

◯ Yes
◯ No

On average how many of the normal structures do you contour in a given treatment plan?

◯ I contour all of the normal structures.
◯ I contour most of the normal structures.
◯ I contour some of the normal structures.
◯ I contour a few of the normal structures.

How confident are you in your ability to accurately contour normal structures?

◯ Extremely confident
◯ Quite confident
◯ Moderately confident
◯ Slightly confident
◯ Not at all confident

How confident are you in your ability to accurately contour each of the following normal structures?

	Extremely confident	Quite confident	Moderately confident	Slightly confident	Not at all confident	I do not routinely contour this normal structure.
Bladder	○	○	○	○	○	○
Brachial Plexus	○	○	○	○	○	○
Brainstem	○	○	○	○	○	○
Cauda Equina	○	○	○	○	○	○
Cochlea	○	○	○	○	○	○
Chest Wall	○	○	○	○	○	○
Duodenum	○	○	○	○	○	○
Esophagus	○	○	○	○	○	○
Eye	○	○	○	○	○	○
Femoral Head	○	○	○	○	○	○
Great Vessels	○	○	○	○	○	○
Heart	○	○	○	○	○	○
Kidney	○	○	○	○	○	○
Large Bowel	○	○	○	○	○	○
Lens	○	○	○	○	○	○
Liver	○	○	○	○	○	○
Lung	○	○	○	○	○	○
Mandible	○	○	○	○	○	○
Optic Chiasm	○	○	○	○	○	○
Optic Nerve	○	○	○	○	○	○
Ovary	○	○	○	○	○	○
Proximal Bronchial Tree	○	○	○	○	○	○
Parotid Gland	○	○	○	○	○	○
Penile Bulb	○	○	○	○	○	○
Prostate	○	○	○	○	○	○
Rectum	○	○	○	○	○	○
Ribs	○	○	○	○	○	○
Sacral Plexus	○	○	○	○	○	○
Small Bowel	○	○	○	○	○	○
Spinal Cord	○	○	○	○	○	○

Stomach	○	○	○	○	○	○
Submandibular Gland	○	○	○	○	○	○
Uterus	○	○	○	○	○	○

On average how many normal structures do you contour with the assistance of auto-segmentation tools?

◯ I contour all normal structures with the assistance of auto-segmentation tools.
◯ I contour most normal structures with the assistance of auto-segmentation tools.
◯ I contour some normal structures with the assistance of auto-segmentation tools.
◯ I contour a few normal structures with the assistance of auto-segmentation tools.
◯ I do not use auto-segmentation tools to assist in contouring normal structures.

How confident are you in the ability of auto-segmentation tools to accurately contour normal structures?

◯ Extremely confident
◯ Quite confident
◯ Moderately confident
◯ Slightly confident
◯ Not at all confident

Have you received any training in contouring normal structures?

◯ Yes
◯ No

Indicate the types of training you have had in contouring normal structures.
(Check all that apply.)

☐ Formal training as part of the curriculum during my education.
☐ Formal training as part of the curriculum in my post-graduate training/medical physics residency.
☐ Formal training as part of my current job.
☐ Formal training as part of a former job.
☐ Attending a talk on contouring normal structures at a professional society meeting.
☐ Informal "on the job" training as part of my current job.
☐ Informal "on the job" training as part of a former job.
☐ Other

What other types of training having you received in contouring normal structures?

How effective was each of the following types of training that you received contouring normal structures?

	Extremely effective	Quite effective	Moderately effective	Slightly effective	Not at all effective
Formal training as part of the curriculum during my education.	○	○	○	○	○
Formal training as part of the curriculum in my post-graduate training/medical physics residency.	○	○	○	○	○
Formal training as part of my current job.	○	○	○	○	○
Formal training as part of a former job.	○	○	○	○	○
Attending a talk on contouring normal structures at a professional society meeting.	○	○	○	○	○
Informal "on the job" training as part of my current job.	○	○	○	○	○
Informal "on the job" training as part of a former job.	○	○	○	○	○
Other training.	○	○	○	○	○

Are there any types of training in contouring normal structures that you have not received that you feel would be helpful?
(Check all that apply.)

☐ Formal training as part of the curriculum during my pre-clinical education.
☐ Formal training as part of the curriculum in my post-graduate training/medical physics residency.
☐ Formal training as part of my current job.
☐ Attending a talk on contouring normal structures at a professional society meeting.
☐ Informal "on the job" training as part of my current job.
☐ Other

What other types of training in contouring normal structures have you not received that you feel would be helpful?

Would training in contouring normal structures increase your confidence in your ability to accurately contour normal structures?

○ Yes
○ No

What types of training in contouring normal structures do you think would be (or would have been) helpful? (Check all that apply.)

☐ Formal training as part of the curriculum during my pre-clinical education.
☐ Formal training as part of the curriculum in my post-graduate training/medical physics residency.
☐ Formal training as part of my current job.
☐ Attending a talk on contouring normal structures at a professional society meeting.
☐ Informal "on the job" training as part of my current job.
☐ Other

What other types of training in contouring normal structures have you not received that you feel would be/would have been helpful?

How often do attending physicians at your clinic review the normal structure contours in their patients' treatment plans?

◯ Always
◯ Most of the time
◯ Some of the time
◯ Rarely
◯ Never
◯ I do not know
◯ Attending physicians at my clinic contour normal structures on their own

Is there a formal process by which attending physicians review the normal structure contours in their patients' treatment plans at your clinic?

◯ Yes
◯ No
◯ I do not know

Please take a minute to describe the formal process your clinic uses to ensure the attending physician reviews normal structure contours prior to treatment planning: (optional)

How useful do you think a formal process by which attending physicians review normal structure contours in their patients' treatment plans would be?

◯ Extremely useful
◯ Quite useful
◯ Moderate useful
◯ Slightly useful
◯ Not at all useful

When does the attending physician review normal structure contours?
(Check all that apply.)

☐ Prior to the treatment planning process
☐ During the treatment planning process
☐ During review of completed treatment plans
☐ After the patient has begun treatment

When the attending physician makes changes to the normal structure contours how often do they explain the reasoning for the changes to the person who originally entered the contours?

◯ Always
◯ Most of the time
◯ Some of the time
◯ Rarely
◯ Never
◯ I do not know

How confident are you in the thoroughness of the review of normal structure contours performed by attending physicians at your clinic?

◯ Extremely confident
◯ Quite confident
◯ Moderately confident
◯ Slightly confident
◯ Not at all confident

Do you ask the attending physician to review normal structure contours if you are unsure of the accuracy of the contours?

◯ Yes
◯ No

How comfortable are you asking the attending physician to review normal structure contours if you are unsure of the accuracy of the contours?

◯ Extremely comfortable
◯ Quite comfortable
◯ Moderately comfortable
◯ Slightly comfortable
◯ Not at all comfortable

Why do you not ask the attending physician to review normal structure contours if you are unsure of the accuracy of the contours?
(Check all that apply.)

☐ Perception that the attending physician is too busy to review normal structure contours.
☐ Not confident that contours will be reviewed thoroughly by the attending physician.
☐ Do not want to delay the treatment planning process by asking the attending physician to review normal structure contours.
☐ Other

Why do you not ask the attending physician to review normal structure contours if you are unsure of the accuracy of the contours?

Are resident physicians present at your clinic?

◯ Yes
◯ No

How often do resident physicians at your clinic review normal structure contours?

◯ Always
◯ Most of the time
◯ Some of the time
◯ Rarely
◯ Never
◯ I do not know
◯ Resident physicians enter normal structure contours on their own

How confident are you in the thoroughness of the review of normal structure contours performed by resident physicians at your clinic?

◯ Extremely confident
◯ Quite confident
◯ Moderately confident
◯ Slightly confident
◯ Not at all confident

Target Volume Contours and Image Fusion

Who contours target volumes at your clinic?
(Check all that apply.)

☐ Attending physicians
☐ Resident physicians
☐ Dosimetrists
☐ Medical physicists
☐ Radiation therapists
☐ Other

Who else contours target volumes at your clinic?

____

Do you contour target volumes as part of your clinical responsibilities?

◯ Yes
◯ No

How often do you contour target volumes?

◯ I contour target volumes in all of the treatment plans I work on.
◯ I contour target volumes in most of the treatment plans I work on.
◯ I contour target volumes in some of the treatment plans I work on.
◯ I contour target volumes in a few of the treatment plans I work on.

Who performs the fusion of clinical image sets for treatment planning purposes at your clinic?
(Check all that apply.)

☐ Attending physicians
☐ Resident physicians
☐ Dosimetrists
☐ Medical physicists
☐ Radiation therapists
☐ Other

Who else performs the fusion of clinical image sets for treatment planning purposes at your clinic?

____

Do you fuse clinical image sets as part of your clinical responsibilities?

◯ Yes
◯ No

How confident are you in your ability to appropriately fuse clinical image sets?

◯ Extremely confident
◯ Quite confident
◯ Moderately confident
◯ Slightly confident
◯ Not at all confident

How confident are you in your ability to appropriately fuse each of the following imaging modalities with the primary image set that is obtained from the simulation?

	Extremely confident	Quite confident	Moderately confident	Slightly confident	Not at all confident	I do not routinely fuse this imaging modality
CT	○	○	○	○	○	○
MRI	○	○	○	○	○	○
PET	○	○	○	○	○	○
SPECT	○	○	○	○	○	○

On average how many image fusions do you perform with the assistance of automated image fusion software?

◯ I perform all of the image fusions with the assistance of automated image fusion software.
◯ I perform most of the image fusions with the assistance of automated image fusion software.
◯ I perform some of the the image fusions with the assistance of automated image fusion software.
◯ I perform a few of the image fusions with the assistance of automated image fusion software.
◯ I do not use automated image fusion software to assist with image fusion.

How confident are you in the ability of automated image fusion software to accurately perform image fusion?

◯ Extremely confident
◯ Quite confident
◯ Moderately confident
◯ Slightly confident
◯ Not at all confident

How often do attending physicians at your clinic review fused clinical image sets to ensure the fusion is appropriate?

◯ Always
◯ Most of the time
◯ Some of the time
◯ Rarely
◯ Never
◯ I do not know.
◯ Attending physicians fuse their own clinical image sets

When the attending physician makes changes to the image fusion how often do they explain the reasoning for the changes to the person who originally fused the images?

◯ Always
◯ Most of the time
◯ Some of the time
◯ Rarely
◯ Never
◯ I do not know

How confident are you in the thoroughness of the review of fused image sets performed by attending physicians at your clinic?

◯ Extremely confident
◯ Quite confident
◯ Moderately confident
◯ Slightly confident
◯ Not at all confident

Do you ask the attending physician to review fused image sets if you are unsure of the appropriateness of the fusion?

◯ Yes
◯ No

How comfortable are you asking the attending physician to review fused image sets if you are unsure of the appropriateness of the fusion?

◯ Extremely comfortable
◯ Quite comfortable
◯ Moderately comfortable
◯ Slightly comfortable
◯ Not at all comfortable

Why do you not ask the attending physician to review fused image sets if you are unsure of the appropriateness of the fusion?
(Check all that apply.)

☐ Perception that the attending physician is too busy to review normal structure contours.
☐ Not confident that contours will be reviewed thoroughly by the attending physician.
☐ Do not want to delay the treatment planning process by asking the attending physician to review normal structure contours.
☐ Other

Why do you not ask the attending physician to review fused image sets if you are unsure of the appropriateness of the fusion?

Rank the frequency with which the following circumstances contribute to the need to make changes to treatment plans either during the treatment planning process or after the plans have been approved in your clinic from 1 (most frequent) to 5 (least frequent). Please use each number only once.

	1 (most frequent)	2	3	4	5 (least frequent)
Inappropriate fusion of image sets for target volume delineation	○	○	○	○	○
Inaccurate normal structure contours	○	○	○	○	○
Lack of adequate communication between the attending physician and treatment planner	○	○	○	○	○
Lack of thorough review of the initial treatment plan	○	○	○	○	○
Changes to the plan of care as a result of a change to the patient's clinical status (e.g. change in performance status; new site of disease; patient can no longer hold his/her breath, tolerate compression belt, lay flat, lay prone, etc.)	○	○	○	○	○

Under which circumstances are changes made to a treatment plan after the plan has been approved, but before the patient starts treatment in your clinic?
(Check all that apply.)

- ☐ Formal (e.g. regularly scheduled planning rounds, chart rounds, film review, tumor board with other physicians, etc.) peer review discovered issues with the target contours (e.g. with/without nodal coverage)
- ☐ Formal peer review discovered issues with normal structures
- ☐ Formal peer reviewed requested changes to the type of treatment (e.g. IMRT versus 3D, block changes)
- ☐ Informal (ad hoc physician-to-physician discussion) peer review discovered issues with target contours
- ☐ Informal peer review discovered issues with normal structures
- ☐ Informal peer reviewed requested changes to the type of treatment
- ☐ Changes to the plan of care as a result of a change to the patient's clinical status (e.g. change in performance status, new site of disease)
- ☐ Other

Under which other circumstances are changes made to a treatment plan after the plan has been approved, but before the patient starts treatment in your clinic?

Under which circumstances are changes made to a treatment plan after the patient starts treatment in your clinic?
(Check all that apply.)

- ☐ Formal peer review discovered issues with the target contours
- ☐ Formal peer review discovered issues with normal structures
- ☐ Formal peer reviewed requested changes to the type of treatment
- ☐ Informal peer review discovered issues with target contours
- ☐ Informal peer review discovered issues with normal structures
- ☐ Informal peer reviewed requested changes to the type of treatment
- ☐ Changes to the plan of care as a result of a change to the patient's clinical status (e.g. change in performance status; new site of disease; patient can no longer hold his/her breath, tolerate compression belt, lay flat, lay prone, etc.)
- ☐ Other

Under which other circumstances are changes made to a treatment plan after the patient starts treatment in your clinic?
