# Supplementary material for: Physician review of image registration and normal structure delineation
Source: J Appl Clin Med Phys. 2020 Sep 28;21(11):80–7. doi: 10.1002/acm2.13031 (PMC7701106; doi:10.1002/acm2.13031)
Supplement: Supplementary file 2 — File S2. Invitation e‐mail sent to the MEDPHYS and MEDDOS listserv groups with the link to the survey. [file ACM2-21-80-s002.docx]

**SUBJECT:** International Normal Structure, Target Volume, and Fusion Review Survey

We are conducting a survey of clinical practices pertaining to delineation and review of normal structure contours, target volume contours, and image fusion for 3D and IMRT treatment planning. We are specifically surveying dosimetrists and physicists to find out your perception of this critical aspect of clinical practice.

Here is the link to the survey: [International Normal Structure, Target Volume, and Fusion Review Survey](https://redcap.uchicago.edu/surveys/?s=7484LY3RHH)

If the link above does not work, try copying the following URL into your web browser: <https://redcap.uchicago.edu/surveys/?s=7484LY3RHH>

We estimate that this survey will take you approximately 10-15 minutes to complete.

Your responses are anonymous. We appreciate your thoughtful and honest answers.

Questions or comments can be addressed to Dan Golden ([dgolden@radonc.uchicago.edu](mailto:dgolden@radonc.uchicago.edu)). The survey was approved as exempt by the University of Chicago IRB.

The survey will remain open through Friday, 2/15/19, 11:59 pm Eastern Time.

Thank you in advance for taking the time to fill out this survey.

Sincerely,

W. Tyler Turchan, MD, Department of Radiation and Cellular Oncology, University of Chicago

Hania Al-Hallaq, PhD, Department of Radiation and Cellular Oncology, University of Chicago

Ritu Arya, MD, Department of Radiation and Cellular Oncology, University of Chicago

Jay Burmeister, PhD, Department of Oncology (Radiation Oncology), Wayne State University

Michael Dominello, DO, Department of Oncology (Radiation Oncology), Wayne State University

Robert Hight, BS, MS, Department of Radiation and Cellular Oncology, University of Chicago

Dan Joyce, CMD, Department of Radiation and Cellular Oncology, University of Chicago

Bradley McCabe, PhD, Department of Radiation and Cellular Oncology, University of Chicago

Anne McCall, MD, Department of Radiation and Cellular Oncology, University of Chicago

Eugenia Perevalova, DMP, Department of Radiation and Cellular Oncology, University of Chicago

Christopher Stepaniak, PhD, Department of Radiation and Cellular Oncology, University of Chicago

Kamil Yenice, PhD, Department of Radiation and Cellular Oncology, University of Chicago

Dan Golden, MD, MHPE, Department of Radiation and Cellular Oncology, University of Chicago
