# Supplementary material for: Physician review of image registration and normal structure delineation
Source: J Appl Clin Med Phys. 2020 Sep 28;21(11):80–7. doi: 10.1002/acm2.13031 (PMC7701106; doi:10.1002/acm2.13031)
Supplement: Supplementary file 3 — Caption [file ACM2-21-80-s003.docx]

**Supplemental File 1**: Survey distributed to medical physicists and dosimetrists.

**Supplemental File 2**: Invitation e-mail sent to the MEDPHYS and MEDDOS listserv groups with the link to the survey.
